# 

**Published:** 1849-08

**Authors:** 


### CONTENTS

### OF NO. II.

---

### ORIGINAL COMMUNICATIONS.

### ESSAYS AND CASES.

Art. I.—A Lecture on the Determinative Cause of Labor, delivered to his Class, in Lebanon, Tenn., by F. H. Gordon, M. D. - - - - - - 93

Art. II.—Three Cases of Puerperal Peritonitis. Reported by L. M. Lawrence, M. D., of Nashville, Tenn., Resident Pupil at the Bellevue Hospital, New York, - 100

Art. III.—A Case of Nephritis—Lumbo-Renal Abscess—Death—Postmortem Examination. By Dr. E. B. Haskins, of Clarksville, Tenn. - - - 106

Art. IV.—Report of Cases. By B. I. Raphael, M. D., one of the Attending Surgeons to the Louisville Marine Hospital, - - - - - - 110

Art. V.—A Case of Heart Disease. By D. W. Yandell, M. D., of Louisville, Kentucky, - - - 115

Art. VI.—A Case of Cholera, with Remarks. By R. L. Scruggs, M. D., of Germantown, Tenn. - 123

### REVIEWS.

Art. VII.—Anesthesia, or the Employment of Chloroform and Ether in Surgery, Midwifery, etc. etc. By J. T. Simpson, M. D., F. R. S., E. Prof. Midwifery in the University of Edinburg, Physician Accoucheur to the Queen in Scotland, &c. &c. - - - - 129

Art. VIII.—The Maternal Management of Children in Health and Disease. By Thomas Bull, M. D., Member of the Royal College of Physicians, Author of Hints to Mothers, etc. etc. - - - - - - 149

SELECTIONS FROM AMERICAN AND FOREIGN JOURNALS.

On Irregular Malarious Diseases, and their Counteractions by means of Strychnine. By J. P. Kirtland, M.D., Prof. Phys. Diag. and Theo. and Prac. of Med., Cleveland Medical College, - - - - - 153
Observations on the Prevention of Severe Invasions of Scarlatina. By Thomas Carroll, M. D., of Cincinnati, - 159
A New Method of Reducing Dislocations of the Elbow Joint backwards. By M. Maisonneuve, - - 162
Nitrate of Silver in the Treatment of Abscesses. By M. Nonat, 162
The Cholera and other Prevalent Diseases, - - 163
Inhalation of Nitrate of Silver, &c. By Thomas K. Chambers, M. D., Fellow of the Royal College of Physicians, - - - - - - 166
New Method of Treating Urethral Pains following Gonorrhea, 168
On Vinum Sem. Colchici Opiate in Gonorrhea. By Dr. Froinus, - - - - - - - 168
Collodion in Chilblains, - - - - - - 169
A New Physiological Fact, - - - - - 169
Death from a Drop of Laudanum. By H. V. Wooten, M. D., of Lowndesboro', Alabama, - - - 170
On the Treatment of West India Remittents and Intermittents by Quinine. By Dr. D. Blair, Demarara, - - 171
Statistics of the Hospitals and the Medical Profession in Paris, 175

ORIGINAL INTELLIGENCE.

Ohio Medical Convention, - - - - - 177
Cholera—Dr. Bird and his Sulphur Pills, - - - 178
Death of Prof. Blandin, - - - - - 179
Richmond Medical College, Virginia, - - - 179
Progress of Cholera, - - - - - 180
Mortality in Cincinnati. - - - - - - 183
Books Received, - - - - - - 184

